# Production of plumage ornaments among males and females of two closely related tropical passerine bird species

**DOI:** 10.1002/ece3.3000

**Published:** 2017-04-25

**Authors:** Erik D. Enbody, Samantha M. Lantz, Jordan Karubian

**Affiliations:** ^1^ Department of Ecology and Evolutionary BiologyTulane UniversityNew OrleansLAUSA

**Keywords:** birds, electron microscopy, feather structure, *Malurus* fairywrens, plumage color, signal evolution, social selection

## Abstract

The evolution of elaborate secondary sexual traits (i.e., ornaments) is well‐studied in males but less so in females. Similarity in the appearance of ornaments between males and females supports the view that female ornaments arise as a neutral byproduct of selection on male traits due to genetic correlation between sexes, but recent research suggests an adaptive function of female ornaments in at least some contexts. Information on the degree to which production of ornaments differs between the sexes can shed light on these alternative perspectives. We therefore characterized the structural underpinnings of melanin‐based plumage production in males and females of two closely related passerine bird species (genus *Malurus*). Importantly, both ornamented and unornamented phenotypes in each sex are present between these two species, providing an opportunity to test the null expectation of equivalent modes of production in male and female ornamented phenotypes. In *Malurus alboscapulatus*, ornamented females are qualitatively similar to males, but we describe a distinctive ornamented female phenotype that differs from that of males in lacking a blue sheen and in lower feather barbule density. In *M. melanocephalus*, unornamented males and females are also similar in appearance, and we describe a similarity between unornamented phenotypes of males and females in both color and underlying feather barbule structure and pigment composition. Unornamented male *M. melanocephalus* can flexibly transition to the ornamented phenotype in weeks, and we found extreme differences in color and feather structure between these two alternative male phenotypes. These results contradict the idea that female ornaments have evolved in this system following a simple switch to male‐like plumage by demonstrating greater complexity in the production of the ornamented phenotype in males than in females.

## 1 Introduction

There is growing awareness that a comprehensive understanding of sexual selection depends upon better understanding the female perspective as well as that of males, in part because the selective pressures experienced by females may differ from those experienced by males (Amundsen, 2000; Clutton‐Brock, 2007; Rosvall, 2011; Tobias, Montgomerie, & Lyon, 2012). This has spurred renewed interest in the evolutionary history and current adaptive function of secondary sexual traits, or ornaments, and expression between the sexes (Kraaijeveld, Kraaijeveld‐Smit, & Komdeur, 2007; Nordeide, Kekäläinen, Janhunen, & Kortet, 2013; Price & Whalen, 2009). Ornaments may convey information about an individual's health and quality if production of the ornament is physiologically costly (Zahavi, 1975). An understanding of the proximate mechanism for producing ornaments in each sex is an important component of understanding the form and function of ornaments in both sexes. For example, in the lizard *Sceloperus virgatus*, females produce an honest, sex‐specific throat ornament (Weiss, Kennedy, & Bernhard, 2009) which uses a pigment, lacking in males, that is thought to limit trade‐offs with egg development (Weiss, Foerster, & Hudon, 2012). Female *Onthophagus sagittarius* (dung beetles) have horn weaponry that is similar to males, but produced in a different location (Emlen, Marangelo, Ball, & Cunningham, 2005; Simmons & Emlen, 2008), which is associated with competition for ecological resources (rather than competition for mates, as in males; Watson & Simmons, 2010). However, variation in sex‐specific ornaments within and across systems means that a comprehensive explanation for the production and adaptive function of ornaments in both sexes remains incomplete.

In birds, when ornamentation is present in both sexes, the appearances of males and females are often similar (Amundsen & Parn, 2006). The observation that females possess identical or rudimentary forms of male ornaments first motivated the idea that ornaments evolve in females only as a neutral byproduct of selection on males (Darwin, 1871). The genetic correlation model proposed by Lande (1980) suggests that selection on one sex can be strong enough to produce a correlated inheritance of those traits in the other sex in the absence of selection pressures. However, recent research has identified numerous examples of adaptive benefits to female ornamentation (reviewed in Kraaijeveld et al., 2007). An understanding of the mechanisms underlying female ornament production and expression provides an opportunity to assess the degree to which ornaments in females are rudimentary or analogous to those found in males. For example, careful examination has revealed differences between the sexes in subtle features of color (e.g., in colors in the ultraviolet range; Hunt, Bennett, Cuthill, & Griffiths, 1998) and structural components (Shawkey, Estes, Siefferman, & Hill, 2005) that may imply sex‐specific selection pressures (Heinsohn, 2005). However, such studies remain relatively rare, and a better understanding of the proximate sources of color variation can provide important insights into how selection acts on male and female ornaments (Gluckman, 2014; Maia, Rubenstein, & Shawkey, 2013).

The underlying architecture for color production is largely conserved among birds (Prum, 2006; Shawkey, Hauber, Estep, & Hill, 2006), and as a result, there is reason to expect that similarly ornamented phenotypes in each sex follow similar mechanistic pathways (Shawkey et al., 2005). Coloration in bird feathers is produced by pigments, or through the fine scale arrangements of feather materials into nanostructures that selectively scatter light, or both (Eliason, Maia, & Shawkey, 2015; Hill & McGraw, 2006). Melanin is an endogenously produced pigment that is present across all bird taxa (Stoddard & Prum, 2011) and is the basis for black, brown, or gray coloration in feathers (Fox & Vevers, 1960). In contrast, feather structure properties are responsible for white, matte, and iridescent colors (Shawkey et al., 2006). There is evidence that ornament production by each of these mechanisms has associated physiological costs (Hill & McGraw, 2006) and that these costs may vary across mechanisms (e.g., carotenoid pigments, reviewed in Svensson & Wong, 2011; melanin pigments, reviewed in Guindre‐Parker & Love, 2014; structural properties, Keyser & Hill, 1999). In addition, melanin‐based color production can have pleiotropic effects on physiology and behavior (Roulin & Ducrest, 2013). Therefore, plumage of any variety can potentially serve as an honest signal and face associated selective pressures, making plumage a suitable trait for studying ornament evolution in males and females. We ask how mechanisms of production differ between variable phenotypes of both male and female birds. We reason that patterns of similarity between the sexes for ornament production would provide evidence for a conserved underlying mechanism across sexes, whereas exceptions may suggest alternative selection pressures driving ornamentation in males and females.

**Figure 1:**
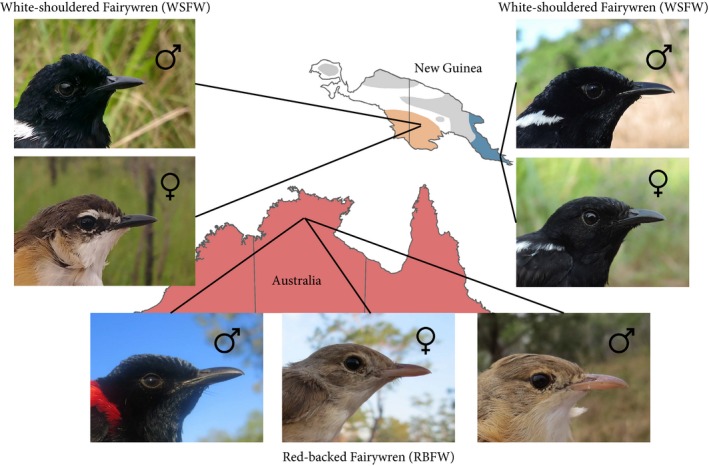
Photographs of the three taxa included in this study and their distributions in New Guinea and Australia. Within the White‐shouldered Fairywren, female crown color is either brown or black in different subspecies, while males remain similarly ornamented black in all subspecies. In Red‐backed Fairywrens, male crown feathers are black in nuptial plumage, but females and males in the nonbreeding season have brown crown feathers. See text for more details. Gray regions of the map refer to other populations of WSFW not included in this study (Rowley & Russell, 1997); ranges based on BirdLife International and Natureserve (2013)

The Australasian *Malurus* fairywrens provide a useful system for studying male and female traits, due to extensive existing research into the behavior, life history, and ecology of the group (Buchanan & Cockburn, 2013) and the considerable intra‐ and interspecific variation in plumage coloration within the group (Johnson, Price, & Pruett‐Jones, 2013; Karubian, 2013). In the current study, we compared the anatomical basis for variation in the melanin‐based color in the crown, a putative plumage ornament (Rowley & Russell, 1997), in three sister lineages in the “bi‐colored” clade of *Malurus* fairywrens (family Maluridae). We examine two subspecies of *M. alboscapulatus* (White‐shouldered Fairywren: WSFW*;* Meyer 1874) and in *M. melanocephalus* (Red‐backed Fairywren: RBFW*;* Latham 1801) that exhibit considerable variation in both male and female ornamentation (Figure 1). Phylogenetic evidence suggests that these lineages are descendent from a monochromatic ornamented ancestor within Maluridae (Driskell et al., 2011; Johnson et al., 2013; Karubian, 2013; Lee, Joseph, & Edwards, 2012) and that female ornamentation was lost in the genus *Malurus* relatively recently (Friedman & Remeš, 2015). For our purposes in the current study, however, the relevant female ancestral state is of an unornamented ancestor at the level of the bi‐colored clade and female ornamentation can be considered a derived character that occurs only in some populations of WSFW following a recent, rapid color change in females (Johnson et al., 2013). Ornamented WSFW populations have been treated as sexually monomorphic in comparative studies (Johnson et al., 2013; Karubian, 2013; but see Friedman & Remeš, 2015), although it has been noted that sexes differ in a “satin sheen” possessed by males and not females (Schodde, 1982). Thus, one open question concerns the degree to which ornamented female WSFW resemble males and, if they differ, what the underlying structural causes of this variation may be. Male RBFW within a population express one of two plumage phenotypes, ornamented or unornamented, and females are unornamented (Karubian, 2002; Rowley & Russell, 1997). Males can flexibly transition from an unornamented to ornamented phenotype within a few weeks (Karubian, Lindsay, Schwabl, & Webster, 2011; Lindsay, Webster, Varian, & Schwabl, 2009). While the ornamented male RBFW is unmistakable, unornamented male and female RBFW are generally indistinguishable in plumage to the human eye; however, experimental evidence suggests that both males and females can distinguish between the two (Karubian, Sillett, & Webster, 2008). Therefore, a second area of inquiry concerns the degree of similarity between dull male and female RBFW, and the structural change in feathers required for males to transition from an unornamented to ornamented state.

We assessed how feather morphology mediates differences in visual signal expression within and among sexes in these two closely related species using photospectrometry and electron microscopy. Our over‐arching null hypothesis was that, across all ornamented sexes and lineages, plumage is produced through similarities in barbule density and fine scale arrangement of melanin in barbules. We find that ornamented plumage in WSFW females is distinct from that of ornamented male WSFW and RBFW. This difference between the ornamented females and males corresponds to differences in feather barbule structure. We also find notable differences in feather morphology (barbule density and melanin content) between ornamented and unornamented male RBFW. In contrast, we found similarity between RBFW unornamented males and unornamented females in color and feather structure. Taken together, these findings demonstrate that the mechanisms of ornament production are similar among males of distinct species, but differ among males and females of a single species. These findings also suggest that that the flexible transition when molting from unornamented to ornamented male phenotypes is associated with substantial structural changes.

## 2 Materials and Methods

### 2.1 Study species and sample collection

Both RBFW and WSFW are socially breeding, tropical, insectivorous passerines that live in grassland environments in Australasia (Rowley & Russell, 1997). The WSFW is endemic to New Guinea, where it is widespread, and the RBFW is endemic to northern and eastern Australia (Rowley & Russell, 1997). Male WSFW are black with a bluish sheen and white shoulder patches and females of the focal populations can either look similar (although perhaps visually duller Schodde, 1982) or are overall brown in color (Figure 1). Ornamentation in some populations of female WSFW is extensive compared to other *Malurus* species (Karubian, 2013) and this intraspecific variation in female ornamentation is rare amongst birds (but see other examples in Bleiweiss, 1992; Andersen et al., 2014; Kearns, White, Austin, & Omland, 2015). Male RBFW are black (with a colorless sheen) with red backs but also have a brown plumage and females are always brown (Figure 1). Male RBFW within a single population exhibit a high degree of flexibility in male plumage development, in contrast to the static interpopulation differences discussed in WSFW above. Most male RBFW molt (pre‐alternate molt) into the black and red ornamented plumage before breeding. First‐year male RBFW can breed as unornamented brown birds (qualitatively similar to females), but are socially subordinate to ornamented individuals (Karubian, 2002; Karubian et al., 2008) and most or all males are unornamented in the nonbreeding season (following the prebasic molt). Female RBFW always molt an unornamented brown plumage (Rowley & Russell, 1997), although a small number of older females (<5%) produce a few red, but not black, feathers (Lindsay, Barron, Webster, & Schwabl, 2016). Both species possess a violet‐sensitive single cone (“SWS1”), meaning they are sensitive to some ultraviolet wavelengths (Ödeen, Hart, & Håstad, 2009; Odeen, Pruett‐Jones, Driskell, Armenta, & Hastad, 2012).

We collected adult crown feathers from 67 ornamented male, 33 ornamented female, and 27 unornamented female WSFW and seven ornamented male, 13 unornamented male, and eight unornamented female RBFW in May‐August, 2014. We collected samples from WSFW for ornamented females in Milne Bay Province, Papua New Guinea (150°30′E,10°15′S, 0–20 m ASL, Figure 1) and for unornamented females from Western Province, Papua New Guinea (141° 19′E, 7° 35′S, 10–20 m ASL, Figure 1). We collected samples from RBFW in Northern Territory, Australia (13°02′S, 131°02′ E, 50 m ASL, Figure 1). We took a small blood sample from each individual and stored red blood cells in lysis buffer for subsequent genetic determination of sex.

### 2.2 Laboratory sexing

To assign sex to unknown individuals, we extracted DNA from blood samples using a DNeasy blood and tissue kit (Qiagen) and amplified a sex‐specific intron within the CHD gene using primers 2550F/2718R (Fridolfsson & Ellegren, 1999). We ran CHD intron fragments through electrophoresis using a 2% agarose minigel and stained with SYBR Safe DNA gel stain (Life Technologies). Bands were scored visually following Kahn, John, and Quinn (1998), using positive controls to confirm accuracy.

### 2.3 Color spectrometry

We used photospectrometry to measure spectral reflectance of all crown feathers. We mounted all sampled crown feathers on black illustration board (Dick Blick Art Materials, Ultra‐black Mounting Board) in an overlapping pattern. We recorded reflectance using an Ocean Optics USB‐2000 + spectrometer (R400‐7‐UV‐VIS probe, RPH‐1 probe holder) with a PX‐2 pulsed xenon light source under laboratory conditions. We recorded % reflectance relative to a WS‐1 white standard (Ocean Optics) for each feather with the probe 7 mm from, and perpendicular to, the surface. Although other angles were investigated, we chose a perpendicular orientation, as we were able to achieve repeatable measurements and observe the saturation of blue sheen of male WSFW feathers (following Shawkey et al., 2006). We used SpectraSuite (Ocean Optics) software to record reflectance curves at 20 scans per sample with an integration time of 100. We averaged three reflectance measurements taken by completely removing the probe and placing back down. We re‐calibrated against the white standard and two color standards at regular intervals to ensure consistency of measurements throughout data collection.

We generated color variables for analysis using the pavo package version 0.5–5 (Maia, Eliason, Bitton, Doucet, & Shawkey, 2013) in R (R version 3.3.0, R Core Team, 2016). To describe achromatic plumage variation and the strength of color signal properties, we calculated brightness as mean reflectance over the entire avian visual spectral range (300–700 nm; Montgomerie, 2006). Low values of brightness represent dark colors and high values represent light colors. We found that hue was not a useful metric to describe chromatic variation in either species (as used in some studies of sexual selection in carotenoid‐based plumage ornaments in birds, e.g., Baldassarre & Webster, 2013), because in the avian tetra color space model, hue is a measure of the horizontal and vertical deviance from the achromatic origin, and our measurements of black/brown feathers were largely clustered around the achromatic origin (Stoddard & Prum, 2008). Instead we examined chroma, which describes the distance a color is from the achromatic origin (Stoddard & Prum, 2008) and is a measure of the relative strength of the plumage color measured (Endler & Mielke, 2005). Chroma is a commonly used metric to describe phenotypic variation and quality of sexual signals (e.g., Cornuault et al., 2015; Doucet, 2004; Shawkey, Estes, Siefferman, & Hill, 2003) and captures variation from blue to black in this species. Chroma was analyzed using the average VS cone‐type retina (Odeen et al., 2012) and idealized illumination in avian tetrahedral color space following Stoddard and Prum (2008).

To compare relative overlap in color between the sexes and phenotypes, we also plotted colors of each sex in tetrahedral color space to represent total color variation of that phenotype (Stoddard & Prum, 2008; Stoddard & Stevens, 2011). We then calculated volume of color space occupied by each sex and present the overlap (relative to the small volume) on a scale of 0–1 to illustrate the overall similarity or difference between sexes following Stoddard and Stevens (2011).

### 2.4 Scanning electron microscopy

We visualized barbule structure using scanning electron microscopy (SEM), which provides a valuable tool for describing the structural component of color production in feathers (Shawkey et al., 2003). We used a subset of feathers from 14 ornamented male, seven ornamented female, and seven unornamented female WSFW and seven ornamented male, six unornamented male, and eight unornamented female RBFW. We mounted individual crown feathers with carbon tape and viewed them using a scanning electron microscope (SEM; Hitachi S4800). We visualized images using ImageJ software (U.S. National Institutes of Health; http://rsb.info.nih.gov/ij/). Differences between the lineages were visible in barbule structure, and we measured barbule structure in two ways. First, following D'Alba et al. (2014), we counted the number of barbules along a 500‐μm transect on the second and third barbs from the distal tip of the feather. Additionally, we measured the density of barbules in a 1 mm^2^ box located at the tip of each crown feather using Image J. Specifically, we used the threshold tool to isolate the feather barbules from the dark background of the image and then measured feather area in a 1 mm^2^ box using the analyze particles tool to give a summary of the percent area of the box that was covered by feather barbules. These two measurements differ in that the first would detect the total number of barbules per barb and the second would detect differences in barbule shape and size.

### 2.5 Transmission electron microscopy

Two crown feathers from each phenotype (Table S1) were embedded for transmission electron microscopy (TEM) following Shawkey et al. (2003). Because of their small size, we prepared and embedded the entire crown feather. We cut barbs using a Leica Reichert Ultracut S microtome and placed sections on 200 mesh copper grids (Ted Pella, Redding CA, USA) with Formvar support, poststained with uranyl acetate, and viewed on a FEI G2 F30 Tecnai TEM (FEI Inc, Hillsboro, OR, USA). Using ImageJ, we compared relevant metrics to melanin and structural colors (Doucet, Shawkey, Hill, & Montgomerie, 2006; Shawkey et al., 2006) including the number of melanin‐containing melanosomes per barbule (“melanosome density”), thickness of the keratin cortex (distance from the outermost melanin granule to the edge of the barbule), and the thickness of the outer layer of melanosomes (distance from the outermost melanin granule to the innermost contiguous melanin granule). Both thickness of the keratin cortex and thickness of the outer layer of melanosomes were averaged across six different points following Maia, D'Alba, and Shawkey (2011).

### 2.6 Statistical analyses

For analysis, males and females were each characterized as possessing either an ornamented (e.g., ornamented male) or unornamented phenotype (e.g., ornamented female). We used a nested analysis of variance (ANOVA) to compare differences in color variables, barbule density, and barbule number (number of barbs per 500 μm) between phenotypes nested within lineage. We compared differences in means between each group using a Tukey Honest Significant Difference test, which corrects for multiple comparisons. Sample sizes for TEM are prohibitively small for statistical analysis, so we present them as tabulated values and qualitative visuals. Brightness measurements were log transformed to achieve homoscedasticity for the above analysis; the other variables had equal variances. Individual linear regressions were performed to test associations between structural properties and color. All analyses were performed in R (R version 3.3.0, R Core Team, 2016), and alpha was set to 0.05.

## 3 Results

Sexes and lineage differed in both brightness and chroma (Table 1, see details below). The number of feather barbules and density of barbules differed between sexes and lineages (Table 1). However, in pairwise comparisons below, only the density of barbules differed suggesting that the shape and structure of barbules, but not overall number of barbules, differed between groups.

**Table 1:** Nested ANOVA results comparing phenotype (e.g., ornamented male, ornamented female, etc.) nested within lineage

	*df*	Sum Sq	Mean Sq	*F*	*p*
Log Brightness: Lineage:Phenotype	4	23.908	5.977	97.210	<.001
Chroma: Lineage:Phenotype	4	0.894	0.223	29.472	<.001
Density: Lineage:Phenotype	4	5186.638	1296.660	41.442	<.001
Num. barbules: Lineage:Phenotype	4	1.480e‐4	3.700e‐05	2.669	.045

### 3.1 Feather ornamentation in White‐shouldered Fairywren (WSFW)

**Figure 2:**
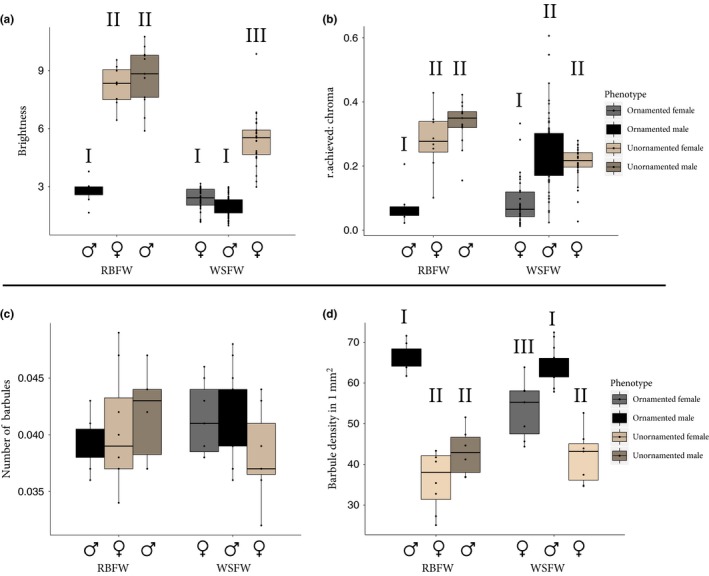
Boxplots for differences in (a) brightness, (b) chroma, (c) number of barbules, and (d) barbule density between lineages and phenotypes. Numerals above each box indicate groups that differ significantly from each other; the same numeral indicates no significant difference. Ornamented female White‐shouldered Fairywrens differ from ornamented male White‐shouldered Fairywren in chroma and barbule density. Unornamented female White‐shouldered Fairywren are less bright than other unornamented phenotypes, but otherwise all unornamented phenotypes are similar

**Figure 3:**
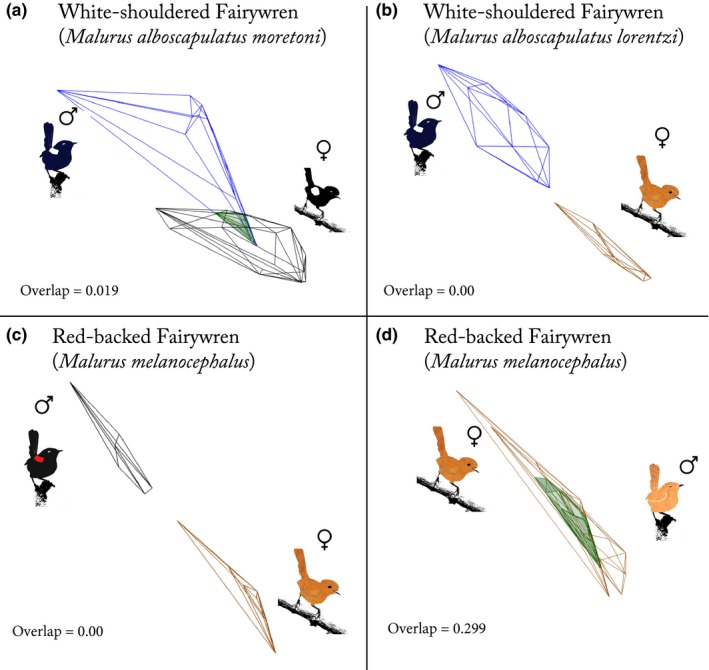
Volume overlap representing plumage color mapped in Cartesian color space to illustrate dichromatism between sexes and phenotypes. Sex and phenotype are illustrated adjacent to their respective polygon and green represents overlap. Images illustrate the separation of color volume occupied between ornamented males and unornamented females (b,c), slight overlap in color between the sexes in the population of White‐shouldered Fairywrens with ornamented females (a), and high overlap between unornamented male and female Red‐backed Fairywrens (d). Overlap between volumes is listed on a scale of 0–1

Ornamented female WSFW were brighter and lower in chroma than unornamented female WSFW, and the same was true when comparing ornamented males to unornamented females (Tables 1 and S1, Figure 2). We also observed lower chroma in ornamented female than ornamented male WSFW, but no difference in brightness (Tables 1 and S1, Figure 2). Ornamented male WSFW did not differ in either measure between the two populations (Figure 2). Using a measure of color space overlap, we found slight overlap between ornamented male and ornamented female WSFW, but no overlap between ornamented male WSFW and unornamented female WSFW (Figure 3).

**Figure 4:**
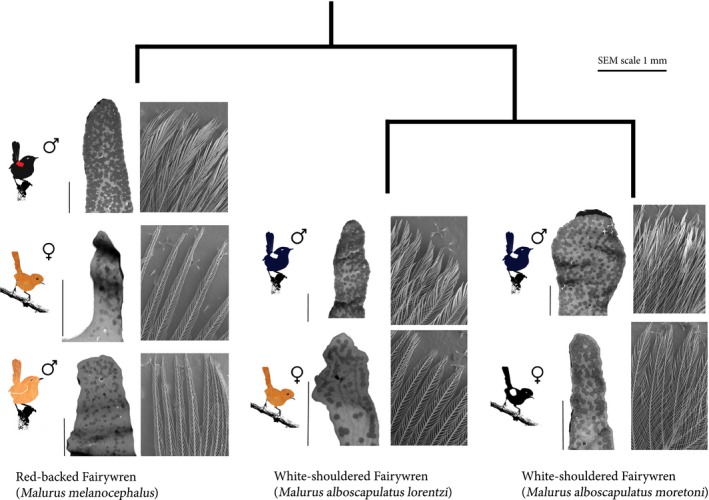
Inferred phylogeny of included lineages based on Driskell et al. (2011) with illustrated phenotypes, SEM, and TEM images of representative crown barbules. Scale bar for all SEM images is shown above, 2μm scale bars for each TEM image is the vertical bar adjacent to each image. Left, note the overall similarity in cross section of melanosome (dark spots) distribution and density between all ornamented phenotypes compared to unornamented phenotypes. Right, note similarity in barbule density between ornamented female White‐shouldered Fairywrens and all unornamented phenotypes, in contrast to the highly dense and clustered ornamented male barbules

These differences in color between lineages were associated with differences in barbule structure in WSFW. In terms of barbule density (via SEM), ornamented males had a greater density of barbules than did ornamented and unornamented females, and there was no difference in number of barbules (Table 1, Figure 2). Overall, ornamented females were intermediate between ornamented males and unornamented females in barbule density (Figures 2 and 4). In cross sections (via TEM), barbules of ornamented male and ornamented females were qualitatively similar in numbers of melanosomes, thickness of the outer melanosome layer, and thickness of the keratin cortex (Table S2, Figure 4). When all ornamented individuals were pooled, chroma was moderately correlated with the thickness of the outer melanosome layer, although this relationship was not significant (*r* ^2^ = .30, *p* = .160). Ornamented females also differed from unornamented females in barbule cross sections by a higher density of melanosomes in each barbule, with a corresponding thick outer layer of melanin and thinner outer keratin layer (Figure 4). With all individuals included, the thickness of the outer keratin layer was positively correlated with brightness (*r* ^2^ = 0.82, *p* < .0001).

### 3.2 Feather ornamentation in Red‐backed Fairywrens (RBFW)

Ornamented males were brighter and lower in chroma than were unornamented male and female RBFW, which were similar to each other (Tables 1 and S1, Figure 2). Ornamented males and unornamented RBFW (of both sexes) overlapped little in color, but unornamented male and female RBFW overlapped to a high degree (Tables 1 and S1, Figure 3).

In terms of barbule density (via SEM), ornamented males had a greater density of barbules than both unornamented males and females, but did not differ in number of barbules (Figures 2 and 4). In cross section (via TEM), barbules in ornamented males had a higher density of melanosomes, a thick outer melanosome layer, and thinner keratin layer compared to both unornamented males and females (ornamented males were similar to that found in male WSFW; Table S2). Unornamented male RBFW were similar to unornamented female RBFW in low number of melanosomes, lacking in a distinct melanosome layer, and a thick keratin layer (Table S2, Figure 4).

## 4 Discussion

A better understanding of the proximal mechanisms underlying ornament production, in combination with related information on phylogeny, behavior, and ecology, has the potential to provide insights into signal evolution and adaptive significance. In the current study on *Malurus* fairywren feather coloration and microstructure, our findings point to an ornamented female *Malurus alboscapulatus* (White‐shouldered Fairywren: WSFW) phenotype that differs from that of males. More specifically, the male ornamented phenotype involves more components (i.e., greater barbule density) than does the female ornamented phenotype. Ornamented plumage in females is recently derived in WSFW (Johnson, Price, & Pruett‐Jones, 2013; above), and these results contradict the idea that female ornamentation was achieved via a simple switch to produce an equivalent ornament to that expressed in males. Moreover, we found that feathers in ornamented male RBFW differ from the unornamented RBFW male plumage both in having a high density of structured melanosomes within barbules and in high barbule density. It is therefore striking that males of this species are able to molt between these alternative plumage states in relatively short time windows. In contrast, we found overall similarity in the color and underlying structure of unornamented males and females of both species.

### 4.1 Feather ornamentation in *Malurus alboscapulatus* (White‐shouldered Fairywren)

Specialized barbule morphology is a widespread mechanism for iridescent plumage in birds (Prum, 2006) and barbule size, shape, and the organization of melanosomes within barbules have also been associated with iridescent color production (Doucet et al., 2006; Maia et al., 2011; Shawkey et al., 2006). Compared to the saturated, blue plumage of male WSFW, the matte black feathers of ornamented female WSFW lack a high density of barbules. The high density of barbules in male WSFW appears to be caused by enlarged and flattened barbules, but not an increase in the number of barbules. This suggests that the production of the blue iridescent sheen in male WSFW is associated with an increased exposure of the nanostructural characteristics found within barbules (as in *Ptilonorhynchus violaceus*, Doucet et al., 2006). In addition, there is a correlative relationship between the thickness of the melanin layer and chroma, and the width of the melanin layer may be involved in how the keratin cortex selectively reflects blue wavelengths (Doucet et al., 2006). In *Volatinia jacarina* (Blue‐black Grassquit), a thin keratin layer over a layer of melanin granules was sufficient to produce a blue sheen (Maia, Caetano, Báo, & Macedo, 2009), and a similar anatomical arrangement may be involved in male WSFW color production. In the absence of these barbule properties, a matte black coloration in ornamented females is produced by the dense melanosome composition of the barbules.

Darwin's (1871) suggestion that ornaments are correlated in their production between the sexes has received both theoretical (Lande, 1980) and empirical support (Potti & Canal, 2011; Price & Pavelka, 1996; Schielzeth, Kempenaers, Ellegren, & Forstmeier, 2012). Due to the similarity in overall patterning between ornamented WSFW of both sexes in the Milne Bay Population, a genetic correlation for ornamentation seems likely for plumage expression in WSFW. However, if female ornaments appear only as a neutral byproduct to selection on males, we should expect they will be identical in form. Our finding that female ornaments in WSFW did not evolve following a simple and immediate switch to male ornamentation indicates that some additional factor is likely involved in the evolution of female ornaments in this species. These findings are consistent with recent research quantifying colors across the family Maluridae that suggests females evolve elaborate colors at different rates and in response to different selective regimes than males (Friedman & Remeš, 2015). However, additional work exploring the selective advantages of female ornamentation will be needed to discern the function of matte black color in this system. Some possibilities include male preference for female ornaments (Amundsen, Forsgren, & Hansen, 1997), a competitive advantage to female ornaments in reproductive (Rubenstein & Lovette, 2009) or ecological contexts, or selection related to survival or nest success (Martin & Badyaev, 1996; Nordeide et al., 2013). Alternatively, matte black color could be selectively neutral, and if producing a bluish sheen incurs costs, natural selection could prevent the evolution of blue in females. Lastly, females may lack the developmental capacity to produce the barbule structure of males, limiting the production of a fully male‐like ornament. Taken together, our results do not rule out a genetic correlation model for explaining the evolution of female ornaments, but they are consistent with sex‐specific selection pressures acting on female ornaments.

Future research might also explore the link between testosterone, feather structure, and the deposition of melanin (Karubian et al., 2011; Lindsay et al., 2009; Peters, 2002; Peters, Astheimer, Boland, & Cockburn, 2000). Testosterone appears to drive acquisition of ornamented plumage in male *Malurus* fairywrens (Lindsay, Webster, & Schwabl, 2011; Peters et al., 2000) and experimental testosterone implants in female *M. cyaneus* produces some male‐like characteristics (without changing color), which could imply a structural change following a rise in testosterone (Peters, 2007). Female RBFW produced carotenoid‐based, but not melanin based, coloration under experimentally elevated testosterone levels (Lindsay et al., 2016). Similarly, preliminary data suggest that unornamented female WSFW produce white feathers, but not melanin‐based black feathers, when testosterone is experimentally elevated (Boersma personal communication). Future work investigating genes that associated with melanin deposition and keratin structure could be informative for describing the underlying mechanism for dichromatism and monomorphism in these groups (San‐Jose et al., 2015; Uy, Moyle, Filardi, & Cheviron, 2009).

### 4.2 Feather ornamentation in *Malurus melanocephalus* (Red‐backed Fairywrens)

In contrast to the WSFW, plumage coloration is similar between unornamented male and female RBFW, as are the underlying mechanisms of sparse feather barbules and randomly distributed melanosomes within barbules. Based on these findings, one might reason that unornamented male RBFW, which during the breeding season are younger individuals (Webster, Varian, & Karubian, 2008), may be mimicking females, a common explanation for delayed plumage maturation (DPM) in birds (Hawkins, Hill, & Mercadante, 2012). However, experimental aviary trials using live birds demonstrate that adult female and male RBFW can distinguish between unornamented male and female RBFW (Karubian et al., 2008). We suggest that conspecifics may be discriminating between young males and females based on behavior or vocalizations, or another body patch such as bill color (Karubian, 2008), or that they are able to perceive differences that do not come up as significant in our analyses. Given the overall similarity in both color and feather structure among unornamented male RBFW and unornamented female RBFW, we propose that genetic correlation between male and female traits likely plays an important role in determining these characteristics.

Although our spectroscopy results suggest similarity in color between ornamented female WSFW and male RBFW, male RBFW have a colorless sheen to their feathers that is visible to the eye (personal observation, Figure 1). The high barbule density is likely involved in the production of this sheen (Doucet et al., 2006; Prum, 2006), as it is the key difference between ornamented males and ornamented females in this study. Future work could focus on how male RBFW and male WSFW produce different colored plumage sheens, which may be the result of different light absorbance in the cortex of the barb rami (Doucet et al., 2006).

Male RBFW transition between unornamented and ornamented plumage between the nonbreeding and breeding seasons, indicating a high degree of flexibility in visual signal development (Karubian, 2002; Karubian et al., 2011; Lantz & Karubian, 2016; Webster et al., 2008). Our work suggests that this transition is achieved by molting in feathers with both higher density of barbules and changes to melanosome deposition. Given that this transition can take place over just a few weeks, it is notable to find overall more structurally complex changes to feathers within male RBFW than between recognized subspecies of female WSFW. The magnitude of this change in structure over such short time periods speaks to the strength of social or sexual selection on male fairywrens.

## 5 Conclusion

By characterizing the structural differences underlying variation in ornamentation among males and females of three closely related lineages of *Malurus* fairywren, we provide insights into the underlying processes driving the evolution of ornament production and sexual dichromatism in this group of birds. We describe an evolutionary transition to ornamentation in WSFW female coloration that is inconsistent with the idea that only genetic correlation between the sexes is responsible for the evolution of female ornaments. Instead, this work implies that female‐specific selection pressures may have driven production of a unique female ornament. These results underscore the importance of explicitly considering the female perspective in evolutionary biology, including work on the mechanistic underpinnings of ornament production. In contrast, we show that the rapid transition (i.e., weeks) from unornamented to ornamented state among male RBFW in response to changes in breeding status involves the greatest degree of structural change we observe in the system, highlighting the relative strength of sexual selection in this highly promiscuous species.

## Conflict of Interest

None declared.
