# Supplementary material for: Production of plumage ornaments among males and females of two closely related tropical passerine bird species
Source: Ecol Evol. 2017 Apr 25;7(11):4024–34. doi: 10.1002/ece3.3000 (PMC5468133; doi:10.1002/ece3.3000)
Supplement: Supplementary file 1 [file ECE3-7-4024-s001.docx]

**Supplementary material**

Table S1: Tukey’s post hoc significance values (p) following a Nested ANOVA.

| Comparison | log_Brightness | Chroma | Density | Num_barbules |
| --- | --- | --- | --- | --- |
| M.a.lorentzi:OM-M.a.moretoni:OF | 0.001 | 0.000 | 0.753 | 0.945 |
| M.a.moretoni:OM-M.a.moretoni:OF | 0.740 | 0.000 | 0.002 | 0.999 |
| M.melanocephalus:OM-M.a.moretoni:OF | 0.911 | 1.000 | 0.006 | 0.994 |
| M.a.lorentzi:UF-M.a.moretoni:OF | 0.000 | 0.000 | 0.015 | 0.907 |
| M.melanocephalus:UF-M.a.moretoni:OF | 0.000 | 0.000 | 0.000 | 1.000 |
| M.melanocephalus:UM-M.a.moretoni:OF | 0.000 | 0.000 | 0.065 | 1.000 |
| M.a.moretoni:OM-M.a.lorentzi:OM | 0.175 | 0.519 | 0.585 | 0.478 |
| M.melanocephalus:OM-M.a.lorentzi:OM | 0.002 | 0.008 | 0.697 | 0.405 |
| M.a.lorentzi:UF-M.a.lorentzi:OM | 0.000 | 1.000 | 0.000 | 0.179 |
| M.melanocephalus:UF-M.a.lorentzi:OM | 0.000 | 0.859 | 0.000 | 0.733 |
| M.melanocephalus:UM-M.a.lorentzi:OM | 0.000 | 0.009 | 0.001 | 0.983 |
| M.melanocephalus:OM-M.a.moretoni:OM | 0.227 | 0.000 | 1.000 | 1.000 |
| M.a.lorentzi:UF-M.a.moretoni:OM | 0.000 | 0.192 | 0.000 | 1.000 |
| M.melanocephalus:UF-M.a.moretoni:OM | 0.000 | 1.000 | 0.000 | 1.000 |
| M.melanocephalus:UM-M.a.moretoni:OM | 0.000 | 0.470 | 0.000 | 0.995 |
| M.a.lorentzi:UF-M.melanocephalus:OM | 0.000 | 0.026 | 0.000 | 1.000 |
| M.melanocephalus:UF-M.melanocephalus:OM | 0.000 | 0.001 | 0.000 | 1.000 |
| M.melanocephalus:UM-M.melanocephalus:OM | 0.000 | 0.000 | 0.000 | 0.983 |
| M.melanocephalus:UF-M.a.lorentzi:UF | 0.001 | 0.648 | 0.724 | 0.990 |
| M.melanocephalus:UM-M.a.lorentzi:UF | 0.000 | 0.002 | 1.000 | 0.852 |
| M.melanocephalus:UM-M.melanocephalus:UF | 1.000 | 0.969 | 0.506 | 1.000 |

Table S2: Tabulated values of measurements of measurements of one barbule cross section under TEM. Num_mel = number of melanosomes; mel.area=number of melanosomes per µm^2^; ker.lay = average width in µm of keratin layer; mel.lay = average width in µm of outer layer of melanosomes.

| Species | Phenotype | num_mel | mel.area | ker.lay | mel.lay |
| --- | --- | --- | --- | --- | --- |
| Red-backed Fairywren (RBFW) | Ornamented male | 527 | 14.466 | 0.0818 | 0.549 |
| Red-backed Fairywren (RBFW) | Ornamented male | 413 | 16.928 | 0.11 | 0.452 |
| Red-backed Fairywren (RBFW) | Unornamented female | 133 | 5.093 | 0.211 | 0.173 |
| Red-backed Fairywren (RBFW) | Unornamented female | 90 | 4.074 | 0.269 | 0.156 |
| Red-backed Fairywren (RBFW) | Unornamented male | 168 | 16.867 | 0.135 | 0.198 |
| Red-backed Fairywren (RBFW) | Unornamented male | 211 | 10.038 | 0.287 | 0.159 |
| White-shouldered Fairywren (WSFW) | Ornamented female | 268 | 14.295 | 0.12 | 0.475 |
| White-shouldered Fairywren (WSFW) | Ornamented female | 205 | 8.812 | 0.108 | 0.528 |
| White-shouldered Fairywren (WSFW) | Ornamented male | 382 | 10.75 | 0.117 | 0.642 |
| White-shouldered Fairywren (WSFW) | Ornamented male | 352 | 8.61 | 0.116 | 0.64 |
| White-shouldered Fairywren (WSFW) | Ornamented male | 719 | 7.938 | 0.0858 | 0.681 |
| White-shouldered Fairywren (WSFW) | Ornamented male | 371 | 12.013 | 0.121 | 0.568 |
| White-shouldered Fairywren (WSFW) | Unornamented female | 249 | 14.102 | 0.159 | 0.162 |
| White-shouldered Fairywren (WSFW) | Unornamented female | 193 | 17.545 | 0.157 | 0.182 |
